# The Impact of Omicron-related Stress on Mental Health in the General Population of China

**DOI:** 10.62641/aep.v53i3.1831

**Published:** 2025-05-05

**Authors:** WenYan Zhao, YuLiang Zhou, YingYing Hu, Jing Wang, Hong Zhu, YaHong Li, ZhiPeng Xu

**Affiliations:** ^1^Department of Neuropsychology, Zhongnan Hospital of Wuhan University, 430000 Wuhan, Hubei, China; ^2^Department of Applied Psychology, South-Central Minzu University, 430000 Wuhan, Hubei, China

**Keywords:** COVID-19, Omicron strain, mental health, resilience, China

## Abstract

**Background:**

Outbreaks of infectious disease represent unique stressors for the general population. In this study, we investigated the prevalence of mental health symptoms and associated risk factors in the general population of China during the Omicron wave.

**Methods:**

We conducted a cross-sectional and large sample online survey during the surge of Omicron cases between 17 December 2022 and 8 January 2023 among Chinese citizens. Then we assessed the prevalence of symptoms of anxiety, depression, insomnia, acute stress disorder, and resilience levels, in the general population of China during the Omicron pandemic by utilizing the coronavirus disease 2019 (COVID-19) anxiety scale (CAS), the Center for Epidemiologic Studies Depression Scale (CES-D), the Insomnia Severity Index scale (ISI), the Stanford Acute Stress Reaction Questionnaire (SASRQ), and the Connor-Davidson Resilience Scale (CD-RISC). Multivariate logistic regression analyses were used to identify demographic and Omicron-related risk factors.

**Results:**

In total, 2800 respondents across 32 provinces and autonomous regions on the Chinese mainland participated in this survey; 1133 (40.5%) were male, and 1860 (66.4%) were 40 years-of-age or younger. The prevalence of anxiety, depression, insomnia, and acute stress disorder was 52%, 58.3%, 45.2%, and 34.8%, respectively. After adjustment for covariates, female gender, a younger age, being unmarried, low income, and a non-medical post were all associated with mental health problems. During the course of infection, participants had a higher risk of developing symptoms of anxiety (odds ratio [OR]: 1.27; 95% confidence interval [CI]: 1.03–1.57; *p* = 0.028), depression (OR: 1.76; 95% CI: 1.44–2.16; *p* < 0.001), insomnia (OR: 1.95; 95% CI: 1.57–2.42, *p* < 0.001) and acute stress disorder (OR: 1.56; 95% CI: 1.25–1.93, *p* = 0.001). In addition, we found that a lower resilience among participants was associated with a higher risk of anxiety, depression, insomnia, and acute stress disorder (*p* < 0.001).

**Conclusion:**

Omicron-related stress had a profound effect on the mental health of the general population of China, especially among those infected during the course of coronavirus disease 2019 (COVID-19) and with lower resilience. Our findings suggest that mental health can be improved during a pandemic by increasing resilience.

## Introduction

Coronavirus disease 2019 (COVID-19) was a pandemic caused by severe acute respiratory syndrome coronavirus 2 (SARS-CoV-2) [1]. According to the World Health Organization (WHO), more than 656 million confirmed cases and more than 6.6 million deaths had been reported worldwide as of 1 January 2019 (WHO). This public health emergency considerably impacted the mental health of the population, resulting in varying degrees of anxiety, depression, insomnia, and other aspects of psychological distress [2, 3]. The mental health issues caused by COVID-19 have gained increasing attention. Early in the pandemic, a study of the general population in China found that patients with COVID-19 had a higher prevalence of mental health symptoms—such as anxiety, depression, suicidal ideation, and insomnia—compared to those who were not infected [4].

Over the past three years, new mutant strains of COVID-19 have emerged. The Omicron variant was first discovered in South Africa in November 2021 [5]. As of 2024, Omicron remains one of the most dominant variants of COVID-19 globally [6]. During the third year of the COVID-19 pandemic, the National Health Commission of China gradually changed the COVID-19 testing strategy in response to the specific epidemiological trend. It is not surprising that the general population is experiencing severe psychological distress in response to this major public health infection [7]. However, there is significant variation in how subjects respond to challenges and difficulties; furthermore, not all infected subjects develop symptoms of anxiety, depression, insomnia, and acute stress disorder. Psychological resilience represents a protective mechanism that can help subjects to maintain a good mental state following traumatic events [8]. However, there has been no specific investigation of how Omicron can result in mental health problems and the resilience of these subjects in the Chinese general population. Therefore, in the present study we conducted an online survey to investigate the psychological distress and factors influencing mental health in the Chinese general population during the Omicron pandemic. Our aim was to provide a theoretical basis and direction for targeted mental health guidance and interventions within the health sector. Our findings highlight the importance of Omicron as a relevant stressor on the psychological status of the general population and will help to understand the complexity of mental health in the context of an epidemic.

## Methods

### Study Design

This cross-sectional online study was conducted between 17 December 2022 and 8 January 2023. Data were collected anonymously through “Survey Star”, an online survey platform managed by Shanghai Changsha Science & Technology. This study was approved by the Ethics Committee of Zhongnan Hospital of Wuhan University (Ref. Number: 2022116K).

### Participants and Data Collection

A total of 3105 respondents participated in the online psychological questionnaire-based survey and provided measures of socio-demographic information and psychological distress (anxiety, depression, sleep state, acute stress disorder, and mental resilience). The inclusion criteria were as follows: (1) Chinese residents living in mainland China during the Omicron epidemic; (2) 16 to 80 years-of-age. Participants who responded for <2 minutes or >30 minutes were excluded to ensure that the questionnaires were good quality. After excluding invalid questionnaires, 2800 participants were included in the analysis.

### Demographic Information

A socio-demographic questionnaire was designed to collect a range of important data from each respondent, including age, gender, marital status, educational level, annual income, residence, chronic medical history, personal and family COVID-19 infection status, post-infection symptoms, working environment, and vaccination status.

### Mental Health Status Scales

The COVID-19 anxiety scale (CAS) was developed to evaluate the specific COVID-19 anxiety caused by the pandemic scenario. Each item is rated on a 4-point scale to reflect the frequency of the symptom, ranging from 0 (not applicable to me) to 3 (very applicable to me) over the past few days. The higher the total score, the higher the level of anxiety relating to COVID-19. Previous studies showed that the seven items of the CAS represent a rapidly administered, valid, and reliable instrument that can be used to measure COVID‑19‑related anxiety [9]. According to the five points of the Generalized Anxiety Disorder-7 (GAD-7) scale (mild degree), the cutoff score is 10 points for the CAS-7 [10].

The Center for Epidemiologic Studies Depression Scale (CES-D) is a widely used instrument for measuring depressive symptoms. This is a self-rating scale that is used to investigate the frequency of depressive symptoms experienced by a subject during the previous week. A total of 10 items were scored on a scale of 0 (little or no) to 3 (most or all of the time) (items 5 and 8 were reverse scores). The suggested cutoff score for clinically probable depression is 10 [11].

The Insomnia Severity Index scale (ISI) is a 7-item self-report assessment for the severity of insomnia symptoms, the impact of sleep interference on daily activities, and the concern caused by sleep problems. Scores of 0–7 are classified as non-clinical insomnia, 8–14 as subclinical insomnia, 15–21 as clinical insomnia (moderate), and 22–28 as clinical insomnia (severe) [12].

The Stanford Acute Stress Reaction Questionnaire (SASRQ) was developed by Cardeña *et al*. [13] to evaluate mental health symptoms in the aftermath of traumatic events. The 30 items of the SASRQ included dissociative symptoms (10 items), continuous re-experience of traumatic events (6 items), avoidance of traumatic events (6 items), high alertness symptoms (6 items), and social function impairment (2 items). Each item is rated on a scale of 6 from 0 to 5. A score of 0 represents “no experience” while a score of 5 represents “always experience” on a scale of 150. A total SASRQ score of ≥40 was used as a criterion for the occurrence of acute stress disorder (ASD); the higher the score, the more severe the ASD symptoms [14]. This study was conducted by psychiatrists specializing in neurology and psychiatry, who diagnosed ASD based on the clinical presentation of a given patient and scoring criteria.

The Connor-Davidson Resilience Scale (CD-RISC) has been widely used and has good reliability and validity in different populations. The 10-item Connor-Davidson Resilience Scale-10 (CD-RISC-10) is an abbreviated CD-RISC version that is used to efficiently measure resilience that yielded excellent psychometric properties when applied in the original English version. Each item is scored on a 5-point scale of 0–4 points (never = 0, rarely = 1, sometimes = 2, often = 3, almost always = 4). The higher the total score, the better the psychological resilience of the subject [15, 16]. This scale lacks a cutoff point; therefore, we divided this scale into three sub-groups: high resilience (score ≥4th quartile), medium resilience (2nd to 4th quartile), and low resilience (score ≤1st quartile) [17].

### Statistical Analysis

Descriptive statistics were used to represent demographic data, with bars showing the prevalence of anxiety, depression, insomnia, and ASD. Univariate analysis of anxiety, depression, insomnia, and ASD using logistic regression model. After controlling for covariates, multivariate logistic regression analysis was used to explain the influencing factors of anxiety, depression, insomnia, and ASD. A two-tailed *p* < 0.05 was considered statistically significant. All statistical analyses were performed using SPSS 25.0 software (IBM, Armonk, NY, USA).

## Results

### Demographic Characteristics

**Fig. 1.**
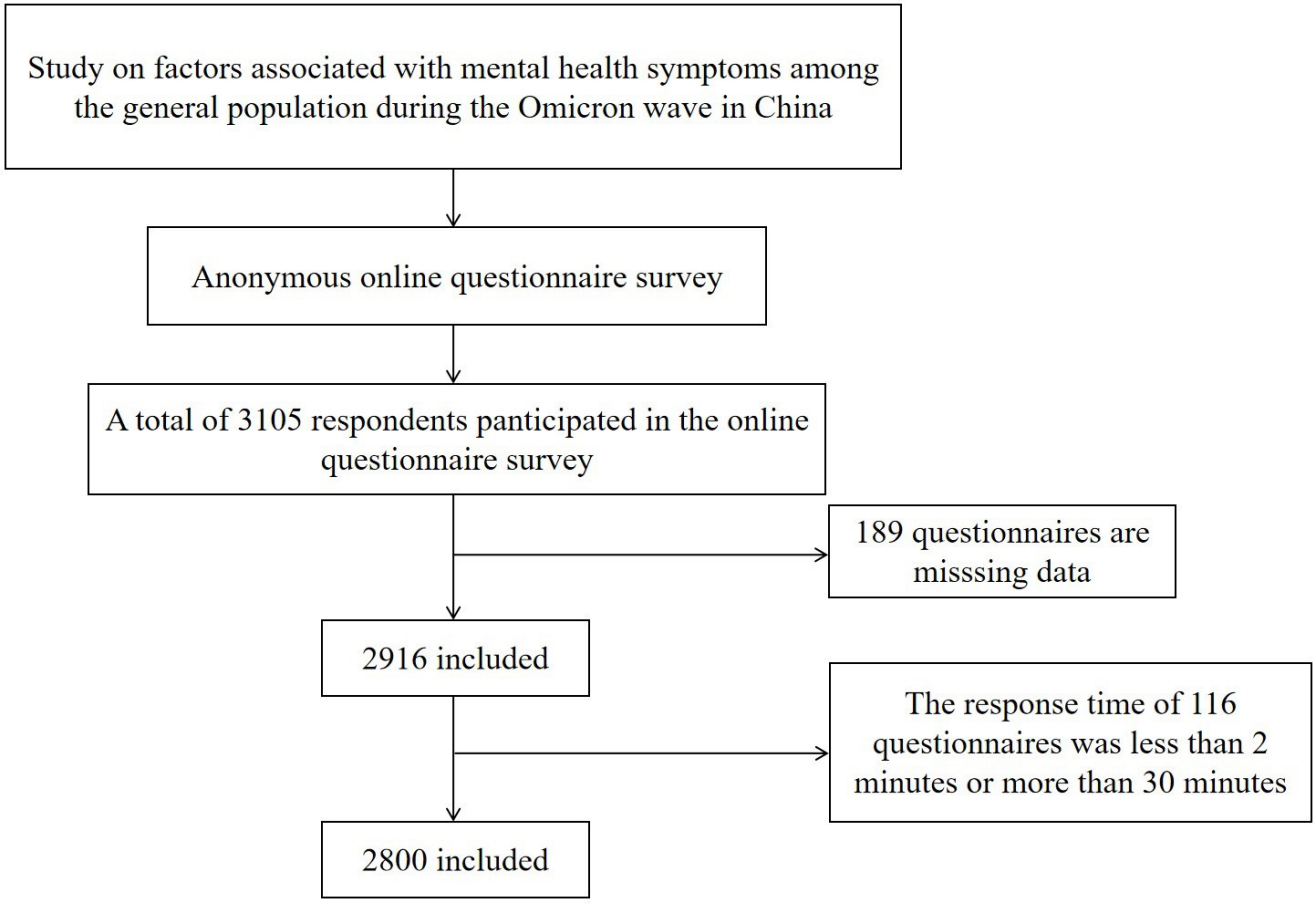
**Flowchart showing how respondents were selected**.

A total of 3105 subjects participated in this survey. In total, 189 were excluded due to missing data; of these, 116 subjects were excluded due to having a response time <2 minutes or >30 minutes. Therefore, 2800 eligible subjects were included in our final analysis, with a valid response rate of 90.2%. Fig. 1 shows a flowchart describing how the final respondents were recruited.

The socio-demographic characteristics are shown in Table 1. Of the 2800 participants, 1667 (59.5%) were female, 940 (33.6%) were over 40 years-of-age, and 1913 (68.3%) were married. Of the 2800 participants, 1480 (52.9%) had a bachelor’s degree, 823 (29.4%) had a master’s degree or above, and 497 (17.8%) had achieved less than a bachelor’s degree. Of the total number of respondents, 1401 (50%) were medical workers and 301 (10.8%) had a history of chronic diseases. In terms of annual income, 662 (23.6%) had an income <CNY 50,000 (1 EUR = 7.6760 CNY), 900 (32.1 %) had an income between CNY 50,000 and 100,000, 809 (28.9%) had an income between CNY 110,000 and 200,000, and 429 (15.3%) had an income above CNY 200,000. According to our analyses, the two most common symptoms of COVID-19 infection were fever and cough; other symptoms included headache, general pain and weakness, and sore throat. In total, 2205 (78.8%) of the respondents had at least one family member infected with COVID-19. Overall, 2607 (93.1%) respondents indicated that there were confirmed or suspected cases in their work environment. The majority of participants (2089 [74.6%]) received three doses of vaccine. Additionally, 672 (24%) were not infected with COVID-19, 1230 (43.9%) were confirmed to be in the course of COVID-19, and 898 (32.1%) had recovered from COVID-19.

**Table 1.** **Demographic characteristics and epidemic-related information from all respondents**.

Factors		Respondents, number (%)
Overall		2800 (100.0)
Gender		
	Male	1133 (40.5)
	Female	1667 (59.5)
Age		
	≤40 years	1860 (66.4)
	>40 years	940 (33.6)
Marital status		
	Unmarried	887 (31.7)
	Married	1913 (68.3)
Level of education		
	Lower than bachelor’s degree	497 (17.8)
	Bachelor’s degree	1480 (52.9)
	Master’s degree or above	823 (29.4)
Occupation		
	Medical workers	1401 (50.0)
	Public institutions or administrative personnel	293 (10.5)
	Commerce occupations	251 (9.0)
	Students	314 (11.2)
	Others	541 (19.3)
Annual income, CNY		
	50,000 or less	662 (23.6)
	50,000–100,000	900 (32.1)
	110,000–200,000	809 (28.9)
	200,000 or more	429 (15.3)
History of chronic diseases		
	Yes	301 (10.8)
	No	2499 (89.3)
What is your current status with COVID-19?		
	Uninfected	672 (24.0)
	In the course of an infection	1230 (43.9)
	Recovered	898 (32.1)
What are your symptoms after COVID-19 infection?		
	Fever	1799 (64.3)
	Headache	1564 (55.9)
	General pain and weakness	1597 (57.0)
	Sore throat	1544 (55.1)
	Nasal congestion and runny nose	1488 (53.1)
	Cough	1817 (64.9)
	Expectoration	1462 (52.2)
	Dyspnea	252 (9.0)
	Diminished or lost sense of taste	853 (30.5)
	Reduced or lost sense of smell	634 (22.6)
Have any of your family members recently been infected with COVID-19?		
	Yes	2205 (78.8)
	No	595 (21.3)
Are there COVID-19 patients in your work environment?		
	Yes	2607 (93.1)
	No	193 (6.9)
How many doses of COVID-19 vaccine have you received?		
	0	66 (2.4)
	1	36 (1.3)
	2	465 (16.6)
	3	2089 (74.6)
	4	144 (5.1)
Anxiety		
	CAS <10	1344 (48.0)
	CAS ≥10	1456 (52.0)
Depression		
	CES-D <10	1169 (41.8)
	CES-D ≥10	1631 (58.3)
Insomnia		
	ISI ≤7	1534 (54.8)
	ISI >7	1266 (45.2)
Acute stress disorder (ASD)		
	SASRQ <40	1825 (65.2)
	SASRQ ≥40	975 (34.8)
Resilience		
	Low resilience (≤1st quartile)	914 (32.6)
	Medium resilience (2nd to 4th quartile)	775 (27.7)
	High resilience (≥4th quartile)	1111 (39.7)

1 EUR = 7.6760 CNY; COVID-19, coronavirus disease 2019; CAS, COVID-19 anxiety scale; CES-D, Center for Epidemiologic Studies Depression Scale; ISI, Insomnia Severity Index scale; SASRQ, Stanford Acute Stress Reaction Questionnaire.

### Prevalence and Risk Factors for Mental Health Symptoms

**Fig. 2.**
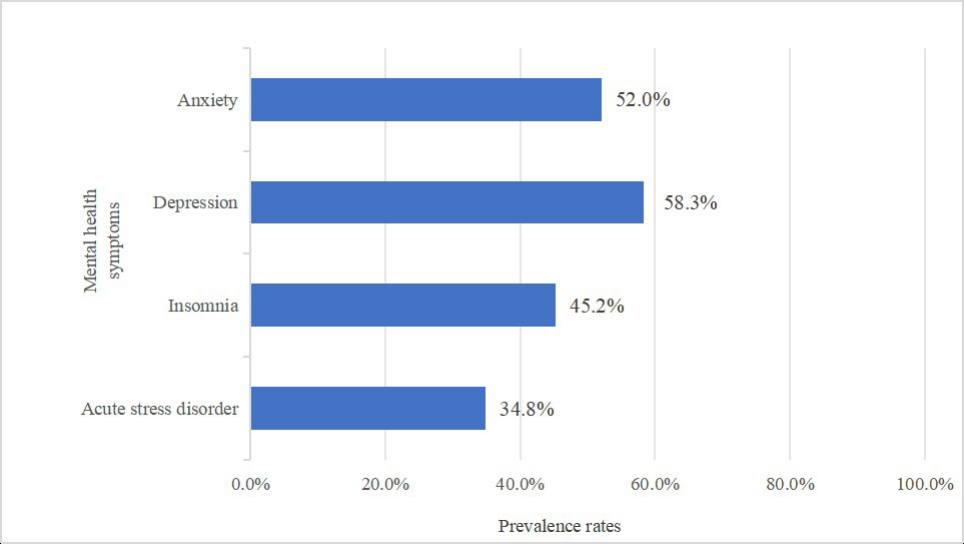
**Prevalence of mental health symptoms in the general population of China during the Omicron pandemic**.

Fig. 2 shows the prevalence of psychological problems in the Chinese population during the epidemic in China. Of the respondents, 52.0% had symptoms of anxiety, 58.3% had symptoms of depression, 45.2% suffered from insomnia, and the prevalence of ASDs was 34.8%.

**Supplementary Tables 1,2,3,4** present the results of our unadjusted analysis for demographic and epidemiological variables among the supplementary data. In multivariate analyses (Tables 2,3,4,5), females (odds ratio [OR]: 1.64; 95% confidence interval [CI]: 1.39–1.94; *p* < 0.001), being a commerce occupations (OR: 1.79; 95% CI: 1.32–2.43; *p* < 0.001), lower income level (OR: 1.80; 95% CI: 1.30–2.50; *p* < 0.001), being in the course of infection (OR: 1.27; 95% CI: 1.03–1.57, *p* = 0.028), and low resilience (OR: 2.33; 95% CI: 1.94–2.81; *p* < 0.001) were identified as risk factors for anxiety. Females (OR: 1.27; 95% CI: 1.07–1.50; *p* = 0.006), aged < 40 years (OR: 1.49; 95% CI: 1.23–1.82; *p* < 0.001), being unmarried (OR: 1.30; 95% CI: 1.04–1.64; *p* = 0.023), being a commerce occupations (OR: 1.72; 95% CI: 1.27–2.32; *p* < 0.001), being in the course of infection (OR: 1.76: 95% CI: 1.44–2.16; *p* < 0.001), and low resilience (OR: 3.28; 95% CI: 2.70–3.98, *p* < 0.001) were identified as risk factors for depression. Being unmarried (OR: 1.28; 95% CI: 1.02–1.60; *p* = 0.031), having a bachelor’s degree (OR: 1.33; 95% CI: 1.10–1.62; *p* = 0.003), being a commerce occupations (OR: 1.52; 95% CI: 1.13–2.05; *p* = 0.006), being in the course of infection (OR: 1.95; 95% CI: 1.57–2.42; *p* < 0.001), and low resilience (OR: 3.38; 95% CI: 2.79–4.09; *p* < 0.001) were identified as risk factors for insomnia. The risk of ASD was significantly higher among commerce occupations (OR: 2.20; 95% CI: 1.60–3.02; *p* < 0.001), lower income levels (OR: 1.94; 95% CI: 1.34–2.79; *p* < 0.001), being in the course of infection (OR: 1.56; 95% CI: 1.25–1.93; *p* = 0.001), and low resilience (OR: 5.64; 95% CI: 4.58–6.96; *p* < 0.001).

**Table 2.** **Multivariate logistic regression analysis of factors related to anxiety during the Omicron variant pandemic**.

Dependent variables	Independent variables	Reference	β	S.E.	Wald	OR	95% CI	*p*
Anxiety	Gender	Male	0.50	0.08	35.23	1.64	1.39–1.94	<0.001
	Age	≥40 years	0.13	0.09	1.84	1.13	0.95–1.36	0.175
	Level of education	Master degree or above						
	Lower than bachelor’s degree		0.11	0.14	0.68	1.12	0.86–1.47	0.410
	Bachelor’s degree		0.04	0.10	0.14	1.04	0.86–1.25	0.713
	Occupation	Medical workers						
	Public institutions or administrative personnel		0.37	0.14	7.13	1.44	1.10–1.88	0.008
	Commerce occupations		0.58	0.16	14.02	1.79	1.32–2.43	<0.001
	Students		−0.09	0.17	0.29	0.91	0.66–1.27	0.593
	Others		0.17	0.11	2.02	1.18	0.94–1.47	0.156
	Annual income, CNY	200,000 or more						
	50,000 or less		0.59	0.17	12.22	1.80	1.30–2.50	<0.001
	50,000–100,000		0.57	0.14	16.87	1.76	1.34–2.30	<0.001
	110,000–200,000		0.49	0.13	14.08	1.64	1.27–2.11	<0.001
	What is your current status with COVID-19?	Uninfected						
	In the course of an infection		0.24	0.11	4.83	1.27	1.03–1.57	0.028
	Recovered		0.15	0.12	1.61	1.16	0.92–1.46	0.204
	Have any of your family members recently been infected with COVID-19?	No	0.13	0.17	0.65	1.14	0.83–1.58	0.420
	How many doses of COVID-19 vaccine have you received?	4						
	0		0.03	0.31	0.01	1.03	0.56–1.91	0.922
	1		0.30	0.40	0.55	1.34	0.62–2.94	0.459
	2		0.15	0.21	0.54	1.16	0.78–1.73	0.464
	3		0.12	0.19	0.40	1.12	0.78–1.62	0.530
	Resilience	High resilience (≥4th quartile)						
	Low resilience (≤1st quartile)		0.85	0.10	78.89	2.33	1.94–2.81	<0.001
	Medium resilience (2nd to 4th quartile)		0.66	0.10	45.70	1.94	1.60–2.35	<0.001

1 EUR = 7.6760 CNY. S.E., Standard Error; OR, odds ratio; CI, confidence interval.

**Table 3.** **Multivariate logistic regression analysis of factors related to depression during the Omicron variant pandemic**.

Dependent variables	Independent variables	Reference	β	S.E.	Wald	OR	95% CI	*p*
Depression	Gender	Male	0.24	0.09	7.59	1.27	1.07–1.50	0.006
	Age	≥40	0.40	0.10	16.26	1.49	1.23–1.82	<0.001
	Marital status	Married	−0.26	0.12	5.14	1.30	1.04–1.64	0.023
	Occupation	Medical workers						
	Public institutions or administrative personnel		0.49	0.14	12.19	1.64	1.24–2.16	<0.001
	Commerce occupations		0.54	0.15	12.34	1.72	1.27–2.32	<0.001
	Students		0.34	0.18	3.83	1.43	1.00–2.05	0.050
	Others		0.09	0.11	0.71	1.10	0.88–1.36	0.399
	Annual income, CNY	200,000 or more						
	50,000 or less		−0.11	0.17	0.44	0.89	0.64–1.25	0.507
	50,000–100,000		−0.17	0.13	1.60	0.85	0.65–1.10	0.205
	110,000–200,000		−0.04	0.13	0.07	0.97	0.75–1.25	0.789
	What is your current status with COVID-19?	Uninfected						
	In the course of an infection		0.57	0.10	29.74	1.76	1.44–2.16	<0.001
	Recovered		0.15	0.11	1.88	1.16	0.94–1.45	0.171
	How many doses of COVID-19 vaccine have you received?	4						
	0		0.22	0.32	0.49	1.25	0.67–2.34	0.483
	1		1.11	0.46	6.01	3.05	1.25–7.43	0.014
	2		0.21	0.21	1.05	1.24	0.83–1.85	0.305
	3		0.19	0.19	1.04	1.21	0.84–1.74	0.308
	Resilience	High resilience (≥4th quartile)						
	Low resilience (≤1st quartile)		1.19	0.10	143.41	3.28	2.70–3.98	<0.001
	Medium resilience (2nd to 4th quartile)		0.79	0.10	63.87	2.21	1.82–2.67	<0.001

1 EUR = 7.6760 CNY.

**Table 4.** **Multivariate logistic regression analysis of factors related to insomnia during the Omicron variant pandemic**.

Dependent variables	Independent variables	Reference	β	S.E.	Wald	OR	95% CI	*p*
Insomnia	Age	≥40 years	0.05	0.10	0.22	1.05	0.86–1.28	0.636
	Marital status	Married	0.25	0.11	4.67	1.28	1.02–1.60	0.031
	Level of education	Master degree or above						
	Lower than bachelor’s degree		0.21	0.14	2.19	1.23	0.94–1.61	0.139
	Bachelor degree		0.29	0.10	8.74	1.33	1.10–1.62	0.003
	Occupation	Medical workers						
	Public institutions or administrative personnel		0.36	0.14	6.95	1.44	1.10–1.88	0.008
	Commerce occupations		0.42	0.15	7.44	1.52	1.13–2.05	0.006
	Students		−0.34	0.18	3.68	0.72	0.51–1.01	0.055
	Others		0.05	0.11	0.20	1.05	0.84–1.32	0.652
	Annual income, CNY	200,000 or more						
	50,000 or less		0.08	0.17	0.24	1.09	0.78–1.52	0.626
	50,000–100,000		0.02	0.14	0.03	1.02	0.78–1.34	0.872
	110,000–200,000		−0.10	0.13	0.02	0.98	0.76–1.27	0.888
	What is your current status with COVID-19?	Uninfected						
	In the course of an infection		0.67	0.11	36.49	1.95	1.57–2.42	<0.001
	Recovered		−0.00	0.12	0.00	1.00	0.79–1.26	0.962
	Have any of your family members recently been infected with COVID-19?	No	0.10	0.11	0.86	1.11	0.89–1.37	0.354
	Resilience	High resilience (≥4th quartile)						
	Low resilience (≤1st quartile)		1.22	0.10	155.54	3.38	2.79–4.09	<0.001
	Medium resilience (2nd to 4th quartile)		0.70	0.10	48.80	2.00	1.65–2.44	<0.001

1 EUR = 7.6760 CNY.

**Table 5.** **Multivariate logistic regression analysis of factors related to acute stress disorder during the Omicron variant pandemic**.

Dependent variables	Independent variables	Reference	β	S.E.	Wald	OR	95% CI	*p*
Acute stress disorder	Gender	Male	0.10	0.09	1.19	1.11	0.92–1.32	0.276
	Age	≥40 years	0.08	0.11	0.48	1.08	0.87–1.33	0.487
	Marital status	Married	0.05	0.12	0.21	1.06	0.84–1.33	0.651
	Level of education	Master degree or above						
	Lower than bachelor’s degree		−0.14	0.15	0.88	0.87	0.65–1.16	0.347
	Bachelor’s degree		0.04	0.11	0.15	1.04	0.85–1.28	0.697
	Occupation	Medical workers						
	Public institutions or administrative personnel		0.36	0.15	5.84	1.43	1.07–1.91	0.016
	Commerce occupations		0.79	0.16	23.40	2.20	1.60–3.02	<0.001
	Students		0.09	0.18	0.26	1.10	0.77–1.56	0.609
	Others		0.20	0.12	2.72	1.22	0.96–1.56	0.099
	Annual income, CNY	200,000 or more						
	50,000 or less		0.66	0.19	12.55	1.94	1.34–2.79	<0.001
	50,000–100,000		0.36	0.15	5.62	1.44	1.07–1.94	0.018
	110,000–200,000		0.26	0.15	3.05	1.30	0.97–1.73	0.081
	What is your current status with COVID-19?	Uninfected						
	In the course of an infection		0.44	0.11	15.97	1.56	1.25–1.93	0.001
	Recovered		0.00	0.12	0.00	1.00	0.79–1.27	0.986
	Resilience	High resilience (≥4th quartile)						
	Low resilience (≤1st quartile)		1.73	0.11	262.57	5.64	4.58–6.96	<0.001
	Medium resilience (2nd to 4th quartile)		0.96	0.09	74.09	2.61	2.10–3.24	<0.001

1 EUR = 7.6760 CNY.

## Discussion

Although being diagnosed with COVID-19 may no longer be viewed as life-threatening, the general population faces multiple stressors during Omicron outbreak. Previous research showed that the COVID-19 pandemic has had a negative impact on mental health in many countries, thus attracting widespread research attention [18, 19]. In the present study, we identified major demographic risk factors associated with mental disorders during the Omicron pandemic. These included being female, younger than 40 years-of-age, unmarried, and having a low income. These findings were consistent with previous findings [20, 21]. As a vulnerable group, medical workers bore a high level of psychological stress due to their occupational exposure risk during the early stages of the COVID-19 epidemic [22, 23, 24]. Furthermore, the prevalence rate of mental disorders was higher than that of non-medical workers [25]. However, opposite trends were observed for occupation; in the present study, we found that public institutions, administrative personnel, and commercial personnel had a higher susceptibility to mental disorders than medical workers during the Omicron epidemic. Our findings were supported by a previous study which confirmed that medical workers presented with lower levels of anxiety, depression, and acute stress than non-medical workers, as determined by assessing mental health of medical workers after one year of the COVID-19 pandemic [26]. This may be attributable to the experience accumulated by the early epidemic prevention work for medical staff, the development of effective coping strategies, and the popularization of vaccination. However, the general public have less social psychological support, personal protection knowledge, and protective equipment [7].

In the context of the Omicron strain, we found that more than half of the respondents exhibited symptoms of anxiety (52.0%) and depression (58.3%), approximately half of the respondents experienced insomnia (45.2%), and one-third had ASD (34.8%); these data were higher than the prevalence reported during the initial outbreak of COVID-19 [27]. Network analysis conducted in February 2020 evaluated the mental health symptoms of the general population in China and found that the prevalence of anxiety, depression, insomnia, and acute stress symptoms was 27.9%, 31.6%, 29.2% and 24.4%, respectively [28]. According to a report by the Chinese Center for Disease Control and Prevention on 25 Jan 2023, the number of COVID-19 infections in China first increased and then decreased from 9 Dec 2022. Our research was undertaken at a stage when the number of COVID-19 infections in China was skyrocketing, and 76% of cases had been artificially confirmed or suspected cases, thus increasing the risk of infection for the whole population and increasing psychological pressure. As a result, more people are suffering from mental health concerns. A meta-analysis previously showed that the prevalence of post-recovery anxiety, depression, and post-traumatic stress disorder was 19%, 20%, and 28%, respectively, among survivors of a 2002–2003 outbreak of severe acute respiratory syndrome coronavirus 2 (SARS-CoV-2) [29, 30]. The long-term psychological disorders observed in the survivors of SARS highlight the potential long-term mental health complications in patients with COVID-19 [31]. The global public health crisis caused by COVID-19 has lasted longer than many of us expected. It is vital that public health authorities should be prepared to prevent the long-term psychological disorders associated with COVID-19 infection.

The respondents in this study who were in the course of COVID-19 infection were more vulnerable to the negative effects of the pandemic. Early in the outbreak, previous studies reported that COVID-19 patients had higher levels of psychological distress than uninfected patients [4, 32]. We consider that these results were mainly due to the fear of death caused by a new mutated strain, various physical discomforts post-infection, uncertainties such as the lack of effective treatment drugs and epidemic prevention materials, as well as a fear of spreading the virus to family members. Therefore, it is important that we pay more attention to this body of subjects in the course of infection, and to provide timely psychological guidance.

Resilience is considered to be an individual’s ability to cope effectively with adversity and defend against the psychological stress caused by traumatic events [33, 34]. The lower the psychological resilience, the higher the risk of anxiety, depression, insomnia, and ASD. A previous study showed that the prevalence of psychological distress was negatively correlated with mental resilience at the peak of the COVID-19 epidemic [35]. Psychological resilience is essential to the ability to cope effectively with difficulty, uncertainty, and change. Previous studies have shown that people who go out more often, exercise more, receive more social support from their family, friends and significant others, and sleep better have higher levels of resilience [36]. Therefore, enhancing the mental resilience of the public during epidemics should be a public health priority to help the population cope more effectively with stress and distress.

### Limitations

There are several limitations to the present study that should be considered. First, the sample size of patients collected was limited, and the survey predominantly targeted Internet users; thus, the sample was not fully representative. Second, the survey did not account for respondents’ prior history of mental disorders. Third, the results reflect only short-term mental health outcomes during the Omicron pandemic. Further studies are now needed to determine the mental health outcomes associated with Omicron infection.

## Conclusion

In the present study, we found that the prevalence of anxiety, depression, insomnia, and acute stress disorder associated with the Omicron strain in China was notable, especially among confirmed or suspected patients during the course of infection. Furthermore, psychological resilience was associated with the risk of psychological disorders. Even three years after the COVID-19 outbreak, its negative impact on people’s mental health remains crucial, suggesting that the long-term effects of stressful events cannot be ignored. Stressful events, such as epidemics, natural disasters, or social upheavals, often subject individuals to considerable psychological stress. Increasing psychological resilience in the public to enable them to cope effectively with these stressors is critical to mitigating the occurrence of mental health problems. Future studies should focus on longitudinal follow-ups to investigate the long-term mental health outcomes associated with COVID-19 and other stressful events. This will help better understand the ongoing effects of such events on individuals’ psychological states and develop strategies to address stress and emotional distress more effectively.

## Data Availability

The datasets used and analysed during the current study are available from the corresponding authors on reasonable request.

## Abbreviations

COVID-19, coronavirus disease 2019; SARS-CoV-2, severe acute respiratory syndrome coronavirus 2; CAS, COVID-19 anxiety scale; CES-D, The Center for Epidemiologic Studies Depression Scale; ISI, Insomnia Severity Index scale; SASRQ, Stanford Acute Stress Reaction Questionnaire; CD-RISC, Connor-Davidson Resilience Scale.

## Supplementary Material

Supplementary material associated with this article can be found, in the online version, at https://doi.org/10.62641/aep.v53i3.1831.
