# Supplementary material for: The Impact of Omicron-related Stress on Mental Health in the General Population of China
Source: Actas Esp Psiquiatr. 2025 May 5;53(3):464–75. doi: 10.62641/aep.v53i3.1831 (PMC12069919; doi:10.62641/aep.v53i3.1831)
Supplement: Supplementary file 1 [file ActEsp-53-3-464-475-s1.docx]

**Supplementary Table 1**. Univariate logistic regression analysis of factors related to anxiety during the omicron variant pandemic.

| Dependent  variables | Independent variables | Reference | β | S.E. | Wald | OR | 95% CI | P |
| --- | --- | --- | --- | --- | --- | --- | --- | --- |
| Anxiety | Gender | Male | 0.59 | 0.08 | 56.78 | 1.80 | 1.54–2.09 | ＜0.001 |
|  | Age | ≥40 | 0.35 | 0.08 | 19.20 | 1.42 | 1.22–1.66 | ＜0.001 |
|  | Marital status | Married | -0.02 | 0.08 | 0.07 | 0.98 | 0.84–1.15 | 0.792 |
|  | Level of education | Master degree or above |  |  |  |  |  |  |
|  | Bachelor’s degree or below |  | 0.49 | 0.12 | 18.27 | 1.63 | 1.31–2.05 | ＜0.001 |
|  | Bachelor’s degree |  | 0.23 | 0.09 | 7.08 | 1.26 | 1.06–1.50 | 0.008 |
|  | Occupation | Medical workers |  |  |  |  |  |  |
|  | Public institutions or administrative personnel |  | 0.23 | 0.13 | 3.28 | 1.26 | 0.98–1.63 | 0.070 |
|  | Commerce occupations |  | 0.35 | 0.14 | 6.41 | 1.42 | 1.08–1.86 | 0.011 |
|  | Students |  | 0.11 | 0.13 | 0.78 | 1.12 | 0.87–1.43 | 0.376 |
|  | Others |  | 0.17 | 0.10 | 2.92 | 1.19 | 0.98–1.45 | 0.088 |
|  | Annual income, CNY | 200,000 or more |  |  |  |  |  |  |
|  | 50,000 or less |  | 0.77 | 0.13 | 36.68 | 2.16 | 1.68–2.77 | ＜0.001 |
|  | 50,000–100,000 |  | 0.78 | 0.12 | 42.34 | 2.19 | 1.73–2.77 | ＜0.001 |
|  | 110,000–200,000 |  | 0.57 | 0.12 | 21.90 | 1.77 | 1.39–2.25 | ＜0.001 |
|  | History of chronic diseases | No | 0.13 | 0.12 | 1.07 | 1.14 | 0.89–1.44 | 0.301 |
|  | What is your current status with COVID-19? | Uninfected |  |  |  |  |  |  |
|  | In the course of an infection |  | 0.35 | 0.10 | 13.23 | 1.42 | 1.18–1.71 | ＜0.001 |
|  | Recovered |  | 0.27 | 0.10 | 7.10 | 1.31 | 1.08–1.61 | 0.008 |
|  | Have any of your family members recently been infected with COVID-19? | No | 0.24 | 0.09 | 6.88 | 1.28 | 1.06–1.53 | 0.009 |
|  | Are there COVID-19 patients in your work environment? | No | 0.14 | 0.15 | 0.90 | 1.15 | 0.86–1.54 | 0.343 |
|  | How many doses of COVID-19 vaccine have you received? | 4 |  |  |  |  |  |  |
|  | 0 |  | 0.28 | 0.30 | 0.88 | 1.32 | 0.74–2.37 | 0.348 |
|  | 1 |  | 0.62 | 0.38 | 2.67 | 1.85 | 0.88–3.88 | 0.103 |
|  | 2 |  | 0.42 | 0.19 | 4.81 | 1.53 | 1.05–2.22 | 0.028 |
|  | 3 |  | 0.37 | 0.17 | 4.50 | 1.45 | 1.03–2.03 | 0.034 |
|  | Resilience | High resilience (≥4th quartile) |  |  |  |  |  |  |
|  | Low resilience (≤1^st^ quartile) |  | 0.92 | 0.09 | 100.09 | 2.50 | 2.09–3.00 | ＜0.001 |
|  | Medium resilience (2^nd^ to 4^th^ quartile) |  | 0.74 | 0.10 | 59.97 | 2.09 | 1.73–2.52 | ＜0.001 |

**Supplementary Table 2**. Univariate logistic regression analysis of factors related to depression during the omicron variant pandemic.

| Dependent  variables | Independent variables | Reference | β | S.E. | Wald | OR | 95% CI | *P* |
| --- | --- | --- | --- | --- | --- | --- | --- | --- |
| Depression | Gender | Male | 0.30 | 0.08 | 14.59 | 1.35 | 1.16–1.57 | ＜0.001 |
|  | Age | ≥40 | 0.65 | 0.08 | 63.23 | 1.91 | 1.63–2.24 | ＜0.001 |
|  | Marital status | Married | 0.56 | 0.09 | 43.33 | 1.75 | 1.48–2.06 | ＜0.001 |
|  | Level of education | Master degree or above |  |  |  |  |  |  |
|  | Bachelor’s degree or below |  | -0.05 | 0.12 | 0.20 | 0.95 | 0.76–1.20 | 0.655 |
|  | Bachelor’s degree |  | 0.07 | 0.09 | 0.60 | 1.07 | 0.90–1.27 | 0.438 |
|  | Occupation | Medical workers |  |  |  |  |  |  |
|  | Public institutions or administrative personnel |  | 0.34 | 0.13 | 6.81 | 1.41 | 1.09–1.83 | 0.009 |
|  | Commerce occupations |  | 0.40 | 0.14 | 7.95 | 1.49 | 1.13–1.97 | 0.005 |
|  | Students |  | 0.70 | 0.14 | 27.07 | 2.02 | 1.55–2.63 | ＜0.001 |
|  | Others |  | 0.12 | 0.10 | 1.43 | 1.13 | 0.93–1.38 | 0.232 |
|  | Annual income, CNY | 200,000 or more |  |  |  |  |  |  |
|  | 50,000 or less |  | 0.57 | 0.13 | 20.16 | 1.77 | 1.38–2.27 | ＜0.001 |
|  | 50,000–100,000 |  | 0.19 | 0.12 | 2.55 | 1.21 | 0.96–1.52 | 0.110 |
|  | 110,000–200,000 |  | 0.12 | 0.12 | 0.98 | 1.13 | 0.90–1.42 | 0.322 |
|  | History of chronic diseases | No | 0.10 | 0.13 | 0.68 | 1.11 | 0.87–1.41 | 0.417 |
|  | What is your current status with COVID-19? | Uninfected |  |  |  |  |  |  |
|  | In the course of an infection |  | 0.54 | 0.10 | 31.13 | 1.72 | 1.42–2.09 | ＜0.001 |
|  | Recovered |  | 0.18 | 0.10 | 3.01 | 1.19 | 0.98–1.46 | 0.083 |
|  | Have any of your family members recently been infected with COVID-19? | No | 0.08 | 0.09 | 0.65 | 1.08 | 0.90–1.30 | 0.421 |
|  | Are there COVID-19 patients in your work environment? | No | -0.22 | 0.16 | 2.09 | 0.80 | 0.59–1.08 | 0.148 |
|  | How many doses of COVID-19 vaccine have you received? | 4 |  |  |  |  |  |  |
|  | 0 |  | 0.42 | 0.30 | 0.98 | 1.53 | 0.85–2.75 | 0.159 |
|  | 1 |  | 1.31 | 0.43 | 9.08 | 3.70 | 1.58–8.67 | 0.003 |
|  | 2 |  | 0.45 | 0.19 | 5.56 | 1.57 | 1.08–2.29 | 0.018 |
|  | 3 |  | 0.39 | 0.17 | 5.03 | 1.47 | 1.05–2.07 | 0.025 |
|  | Resilience | High resilience (≥4th quartile) |  |  |  |  |  |  |
|  | Low resilience (≤1^st^ quartile) |  | 1.26 | 0.10 | 173.46 | 3.52 | 2.92–4.25 | ＜0.001 |
|  | Medium resilience (2^nd^ to 4^th^ quartile) |  | 0.88 | 0.10 | 83.39 | 2.41 | (2.00–2.91) | ＜0.001 |

**Supplementary Table 3**. Univariate logistic regression analysis of factors related to insomnia during the omicron variant pandemic.

| Dependent  variables | Independent variables | Reference | β | S.E. | Wald | OR | 95% CI | *P* |
| --- | --- | --- | --- | --- | --- | --- | --- | --- |
| Insomnia | Gender | Male | 0.08 | 0.08 | 1.06 | 1.08 | 0.93–1.26 | 0.304 |
|  | Age | ≥40 | 0.25 | 0.08 | 9.33 | 1.28 | 1.10–1.50 | 0.002 |
|  | Marital status | Married | 0.23 | 0.08 | 7.67 | 1.26 | 1.07–1.48 | 0.005 |
|  | Level of education | Master degree or above |  |  |  |  |  |  |
|  | Bachelor’s degree or below |  | 0.36 | 0.12 | 9.88 | 1.43 | 1.15–1.80 | 0.002 |
|  | Bachelor’s degree |  | 0.34 | 0.09 | 14.77 | 1.40 | 1.18–1.67 | ＜0.001 |
|  | Occupation | Medical workers |  |  |  |  |  |  |
|  | Public institutions or administrative personnel |  | 0.25 | 0.13 | 3.69 | 1.28 | 1.00–1.65 | 0.055 |
|  | Commerce occupations |  | 0.28 | 0.14 | 4.10 | 1.32 | 1.01–1.73 | 0.043 |
|  | Students |  | -0.03 | 0.13 | 0.05 | 0.97 | 0.76–1.25 | 0.824 |
|  | Others |  | 0.07 | 0.10 | 0.51 | 1.08 | 0.88–1.31 | 0.477 |
|  | Annual income, CNY | 200,000 or more |  |  |  |  |  |  |
|  | 50,000 or less |  | 0.30 | 0.13 | 5.76 | 1.35 | 1.06–1.73 | 0.016 |
|  | 50,000–100,000 |  | 0.30 | 0.12 | 6.49 | 1.35 | 1.07–1.71 | 0.011 |
|  | 110,000–200,000 |  | 0.10 | 0.12 | 0.76 | 1.11 | 0.88–1.41 | 0.384 |
|  | History of chronic diseases | No | 0.34 | 0.12 | 7.83 | 1.41 | 1.11–1.80 | 0.005 |
|  | What is your current status with COVID-19? | Uninfected |  |  |  |  |  |  |
|  | In the course of an infection |  | 0.70 | 0.10 | 50.13 | 2.01 | 1.65–2.43 | ＜0.001 |
|  | Recovered |  | 0.11 | 0.11 | 1.03 | 1.11 | 0.91–1.37 | 0.310 |
|  | Have any of your family members recently been infected with COVID-19? | No | 0.24 | 0.09 | 6.75 | 1.28 | 1.06–1.53 | 0.008 |
|  | Are there COVID-19 patients in your work environment? | No | 0.21 | 0.15 | 1.92 | 1.23 | 0.91–1.66 | 0.166 |
|  | How many doses of COVID-19 vaccine have you received? | 4 |  |  |  |  |  |  |
|  | 0 |  | 0.49 | 0.30 | 2.64 | 1.63 | 0.91–2.92 | 0.104 |
|  | 1 |  | 0.48 | 0.37 | 1.62 | 1.61 | 0.77–3.35 | 0.203 |
|  | 2 |  | 0.22 | 0.19 | 1.33 | 1.25 | 0.86–1.83 | 0.249 |
|  | 3 |  | 0.16 | 0.18 | 0.82 | 1.17 | 0.83–1.65 | 0.365 |
|  | Resilience | High resilience (≥4^th^ quartile) |  |  |  |  |  |  |
|  | Low resilience (≤1^st^ quartile) |  | 1.23 | 0.09 | 176.17 | 3.48 | 2.89–4.18 | ＜0.001 |
|  | Medium resilience (2^nd^ to 4^th^ quartile) |  | 0.72 | 0.10 | 54.36 | 2.04 | 1.69–2.47 | ＜0.001 |

**Supplementary Table 4**. Univariate logistic regression analysis of factors related to acute stress disorder during the omicron variant pandemic.

| Dependent  variables | Independent variables | Reference | β | S.E. | Wald | OR | 95% CI | *P* |
| --- | --- | --- | --- | --- | --- | --- | --- | --- |
| Acute stress disorder | Gender | Male | 0.19 | 0.08 | 0.53 | 1.21 | 1.03–1.42 | 0.021 |
|  | Age | ≥40 | 0.44 | 0.09 | 25.50 | 1.55 | 1.31–1.84 | ＜0.001 |
|  | Marital status | Married | 0.39 | 0.08 | 22.00 | 1.48 | 1.26–1.75 | ＜0.001 |
|  | Level of education | Master degree or above |  |  |  |  |  |  |
|  | Bachelor’s degree or below |  | 0.27 | 0.12 | 5.00 | 1.31 | 1.03–1.65 | 0.025 |
|  | Bachelor’s degree |  | 0.22 | 0.19 | 5.37 | 1.24 | 1.03–1.49 | 0.021 |
|  | Occupation | Medical workers |  |  |  |  |  |  |
|  | Public institutions or administrative personnel |  | 0.19 | 0.14 | 1.92 | 1.21 | 0.93–1.57 | 0.166 |
|  | Commerce occupations |  | 0.46 | 0.14 | 10.51 | 1.58 | 1.20–2.06 | 0.001 |
|  | Students |  | 0.53 | 0.13 | 17.16 | 1.70 | 1.32–2.18 | ＜0.001 |
|  | Others |  | 0.17 | 0.11 | 2.55 | 1.19 | 0.96–1.46 | 0.110 |
|  | Annual income, CNY | 200,000 or more |  |  |  |  |  |  |
|  | 50,000 or less |  | 0.86 | 0.14 | 39.86 | 2.36 | 1.81–3.08 | ＜0.001 |
|  | 50,000–100,000 |  | 0.51 | 0.13 | 15.07 | 1.66 | 1.29–2.15 | ＜0.001 |
|  | 110,000–200,000 |  | 0.29 | 0.14 | 4.65 | 1.34 | 1.03–1.74 | 0.031 |
|  | History of chronic diseases | No | 0.10 | 0.13 | 0.63 | 1.11 | 0.86–1.42 | 0.428 |
|  | What is your current status with COVID-19? | Uninfected |  |  |  |  |  |  |
|  | In the course of an infection |  | 0.43 | 0.10 | 17.58 | 1.54 | 1.26–1.88 | ＜0.001 |
|  | Recovered |  | 0.05 | 0.11 | 0.21 | 1.05 | 0.85–1.31 | 0.646 |
|  | Have any of your family members recently been infected with COVID-19? | No | 0.18 | 0.10 | 3.46 | 1.20 | 0.99–1.46 | 0.063 |
|  | Are there COVID-19 patients in your work environment? | No | -0.28 | 0.15 | 3.39 | 0.76 | 0.56–1.02 | 0.065 |
|  | How many doses of COVID-19 vaccine have you received? | 4 |  |  |  |  |  |  |
|  | 0 |  | 0.55 | 0.31 | 3.16 | 1.73 | 0.95-3.17 | 0.075 |
|  | 1 |  | 0.16 | 0.40 | 0.16 | 1.17 | 0.54-2.56 | 0.686 |
|  | 2 |  | 0.30 | 0.21 | 2.16 | 1.35 | 0.90-2.03 | 0.142 |
|  | 3 |  | 0.22 | 0.19 | 1.32 | 1.24 | 0.86-1.79 | 0.251 |
|  | Resilience | High resilience (≥4^th^ quartile) |  |  |  |  |  |  |
|  | Low resilience (≤1^st^ quartile) |  | 1.78 | 0.10 | 294.33 | 5.93 | 4.84-7.27 | ＜0.001 |
|  | Medium resilience (2^nd^ to 4^th^ quartile) |  | 1.02 | 0.11 | 87.23 | 2.77 | 2.24-3.43 | ＜0.001 |
